# Noncoding RNAs and Stem Cell Function and Therapy

**DOI:** 10.1155/2018/7306034

**Published:** 2018-06-05

**Authors:** Yaoliang Tang, Wei Lei, Yanfang Chen, Xiaolong Wang, Mark W. Hamrick, Mi Zhou

**Affiliations:** ^1^Medical College of Georgia, Augusta University, Augusta, GA, USA; ^2^Laboratory of Cardiovascular Diseases, Guangdong Medical University, Zhanjiang 524001, China; ^3^Department of Pharmacology & Toxicology, Boonshoft School of Medicine, Wright State University, Dayton, OH 45435, USA; ^4^Cardiovascular Department, Cardiovascular Research Institute of Traditional Chinese Medicine, Shuguang Hospital, Shanghai University of Traditional Chinese Medicine, Shanghai 201203, China; ^5^Department of Cardiac Surgery, Ruijin Hospital, Shanghai Jiao Tong University, Shanghai, China

Noncoding RNAs (ncRNAs), including small microRNAs (miRNAs) and long noncoding RNAs (lncRNAs), are responsible for fine regulation of gene expression in stem cells [1]. This special issue focuses on the two main ncRNAs currently under investigation, miRNAs and lncRNAs, the latter of which are ill-understood compared to miRNAs.

miRNAs are a family of endogenous noncoding RNA molecular about 22 nucleotide in length [2, 3]. Mature miRNAs can mediate translational repression through miRNA-induced silencing complexes that bind to the 3′-untranslated region (3′UTR) of the target mRNA. During mouse embryonic stem cell (mESC) differentiation, many miRNAs are either upregulated or downregulated. Z.-Y. Chen et al. performed a miRNA array screen, and identified miR-142-3p significantly downregulated during mESC differentiation into the mesodermal and cardiac progenitor cells, they did miR-142-3p overexpression and inhibition experiments, and found that regulation of miR-142-3p level does not change the characteristics of undifferentiated mESCs; however, ectopic expression of miR-142-3p inhibits the expression of cardiac transcription factor Mef2c by targeting 3′-untranslated region of Mef2c, suggesting an important regulatory role of miR-142-3p in early cardiac differentiation.

Notch signaling pathway is evolutionarily conserved from invertebrates to vertebrates, Notch signaling is activated via juxtacrine binding of an adjacent cell's Jagged ligands (Jag1 and 2) or Delta-like ligands (Dll1, 3, and 4) with 4 Notch receptors (Notch 1, 2, 3, and 4), Notch signaling pathway directly controls stem cell survival, proliferation, and differentiation [1, 4]. C. Chen et al. investigated the role and mechanism of microRNA-1 (miR-1) in the differentiation of adipose-derived stem cells (ASCs) into cardiomyocyte-like cells. They found that miR-1 could promote the differentiation of ASCs in the myocardial microenvironment, and Notch/Hes1 signaling is involved in ASC differentiation into cardiomyocytes.

Adult cardiomyocytes (CM) have limited proliferative capacity; therefore, stimulating CM proliferation becomes a promising strategy for inducing cardiac regeneration. Noncoding RNAs were found differently expressed in CMs with different proliferation potential. Modulating noncoding RNAs might be a potential strategy to promote adult CM proliferation. S. Qu et al. reviewed the microRNAs which were proved to promote or suppress CM proliferation and the underlying mechanism of miRNA-mediated CM proliferation.

Recent studies proved that the beneficial effect of MSC in cardioprotection is contributed to paracrine effect. Y. Zhou et al. tried to explore the major factors which account for the beneficial effects of MSC; they identified that hepatoma-derived growth factor (HDGF) was one of the important factor secreted by MSCs but not by cardiac fibroblast. Knockdown of HDGF can ablate the cellular protective effect of conditioned medium (CdM) from MSC. Furthermore, they found HDGF-mediated cellular protection is protein kinase C epsilon (PKC*ε*) dependent.

Stem cells can secrete exosomes/microvesicles (30–150 nm), which shuttle miRNAs between cells, and play an important role in miRNA communication between donor stem cells and recipient tissues [5–7]. Exosomes containing biological active miRNAs mediate paracrine effect of mesenchymal stem cells (MSC), and exosome membrane protect miRNAs from RNase degradation. A. Luarte et al. reviewed the latest progress regarding the impact of stress in the biology of the neurogenic niche, especially how exosomes mediate communication between astrocytes and niche cells via exosomes. Tumor-derived exosomes can induce mesenchymal stem cell (MSC) transformation into cancer-associated fibroblast (CAF). Q. Cheng et al. investigated the effects of multiple myeloma- (MM-) derived exosomes on regulating the proliferation of MSC, CAF transformation of MSC, and IL-6 secretion of MSCs; they found that miR-21 and miR-146a from MM derived exosomes play an important role in regulating MSC transformation and cytokine secretion.

lncRNAs are noncoding RNAs that are longer than 200 nucleotides in length that cover the largest and most diverse group of ncRNAs. lncRNAs regulate stem cell potency and differentiation [8]. S. Lee et al. reviewed the major lncRNAs involved in the transcriptional and epigenetic regulation of stem cell differentiation and maintenance. The mechanisms of cytoplasmic lncRNAs and nucleus lncRNAs are different, particularly, cytoplasmic lncRNAs regulate turnover, translation, and silence of partially complementary mRNAs; they can also act as a miRNA sponge to reduce miRNA availability and can modulate signaling pathways via interaction with signaling molecular. Nuclear lncRNAs can be decoys for transcription factors, or serve as a scaffold for ribonucleoprotein (RNP) or serve as an epigenetic regulator by recruiting chromatin modification factors.

3,4-Benzopyrene (Bap) is an important component of cigarette smoke and automobile exhaust. Bap is one of the leading risk factor of abdominal aortic aneurysm (AAA). Macrophage activation plays a key role for Bap-induced AAA; however, the mechanism is unclear. Y. Zhou et al. used a mouse lncRNAs array to investigate the expression signatures of lncRNAs and mRNAs in Bap-activated macrophage. They found that 8 pathways associated with inflammation were upregulated, particularly, the AGE-RAGE pathway, which is involved in Bap-induced dysfunction of endothelial progenitor cell (EPC). This study provides potential targets for AAA caused by smoking.

Endothelial dysfunction is an early step in neointima formation, L. Lv et al. used RNA-sequencing (RNA-seq) to analyze the expression profiles of lncRNAs in human stenosed and nonstenotic uremic veins. They identified unannotated lncRNAs, uc001pwg.1, which was one of the most significantly downregulated lncRNAs. Further studies revealed that uc001pwg.1 overexpression could increase nitric oxide synthase (eNOS) phosphorylation and nitric oxide (NO) production in endothelial cells (ECs). Mechanistically, uc001pwg.1 improves endothelial function via mediating MCAM expression. This study represents the first effort of identifying a novel attractive target for improving arteriovenous fistula (AVF) function in uremic patients.

*Yaoliang Tang* *Wei Lei* *Yanfang Chen* *Xiaolong Wang* *Mark W. Hamrick* *Mi Zhou*
